# Bladder cancer invasion along a tension‐free vaginal mesh

**DOI:** 10.1002/iju5.12254

**Published:** 2021-01-21

**Authors:** Atsuhiko Ochi, Shunsuke Harada, Wataru Fukuokaya, Koichi Honma, Tingwen Huang, Hirokazu Abe

**Affiliations:** ^1^ Departments of Urology Kameda Medical Center Kamogawa Chiba Japan; ^2^ Department of Diagnostic Pathology Kameda Medical Center Kamogawa Chiba Japan

**Keywords:** bladder cancer, carcinoma *in situ*, laparoscopic radical cystectomy, pelvic organ prolapse, tension‐free vaginal mesh

## Abstract

**Introduction:**

The effect of synthetic mesh after pelvic organ prolapse surgery on the progression of bladder cancer remains unclear.

**Case presentation:**

A 79‐year‐old woman who underwent a tension‐free vaginal mesh procedure 8 years prior was diagnosed with carcinoma *in situ* of the bladder. Although intravesical Bacillus Calmette–Guérin therapy was started, the tumor rapidly became muscle invasive. Laparoscopic radical cystectomy was performed following radiochemotherapy; however, the tumor extended to the left internal obturator muscle along the mesh arm. Pathological findings showed desmoplastic high‐grade urothelial carcinoma infiltrating around the mesh. Finally, cancer recurred rapidly in the left internal obturator muscle.

**Conclusion:**

Synthetic mesh can become an abnormal anatomical pathway for tumor infiltration. Therefore, in high‐risk bladder cancer patients who underwent a tension‐free vaginal mesh procedure, radical cystectomy should be performed without delay before the tumor invades the perivesical tissue.

**Abbreviations & Acronyms**
BCG: Bacillus Calmette–Guérin
CIS: carcinoma *in situ*
CT: computed tomography
LRC: laparoscopic radical cystectomy
LSC: laparoscopic sacrocolpopexy
MRI: magnetic resonance imaging
POP: pelvic organ prolapse
TVM: tension‐free vaginal mesh

**Keynote message**

We report a rare case of bladder cancer invading the pelvic wall muscle along the TVM arm. LRC was performed, and pathological findings showed desmoplastic high‐grade urothelial carcinoma infiltrating around the mesh. Synthetic mesh can become an abnormal anatomical pathway for tumor infiltration.

## Introduction

The TVM procedure is commonly used for POP.^1^, ^2^ A synthetic mesh is placed on the anterior and/or posterior aspect of the vagina, suspending the pelvic organ via a transvaginal procedure.^3^ Although LSC has recently become the gold standard,^4^, ^5^ the TVM procedure remains a valid technique in cases contraindicated for LSC or in recurrent cases of native tissue repair for cystocele.

CIS of the bladder is a flat non‐muscle invasive urothelial carcinoma usually treated with intravesical BCG therapy; however, it is a poorly differentiated tumor and can progress aggressively.^6^ CIS of the bladder progress to muscle‐invasive cancer in approximately 54% of patients without any treatment and approximately 10–66% if intravesical treatment with BCG or chemotherapy is performed.^7^

The effect of synthetic mesh after surgery for POP on bladder cancer progression has not been described. We report a rare case of muscle‐invasive bladder cancer that progressed from CIS and invaded the pelvic wall along the mesh arm of the TVM procedure.

## Case presentation

**Fig. 1:**
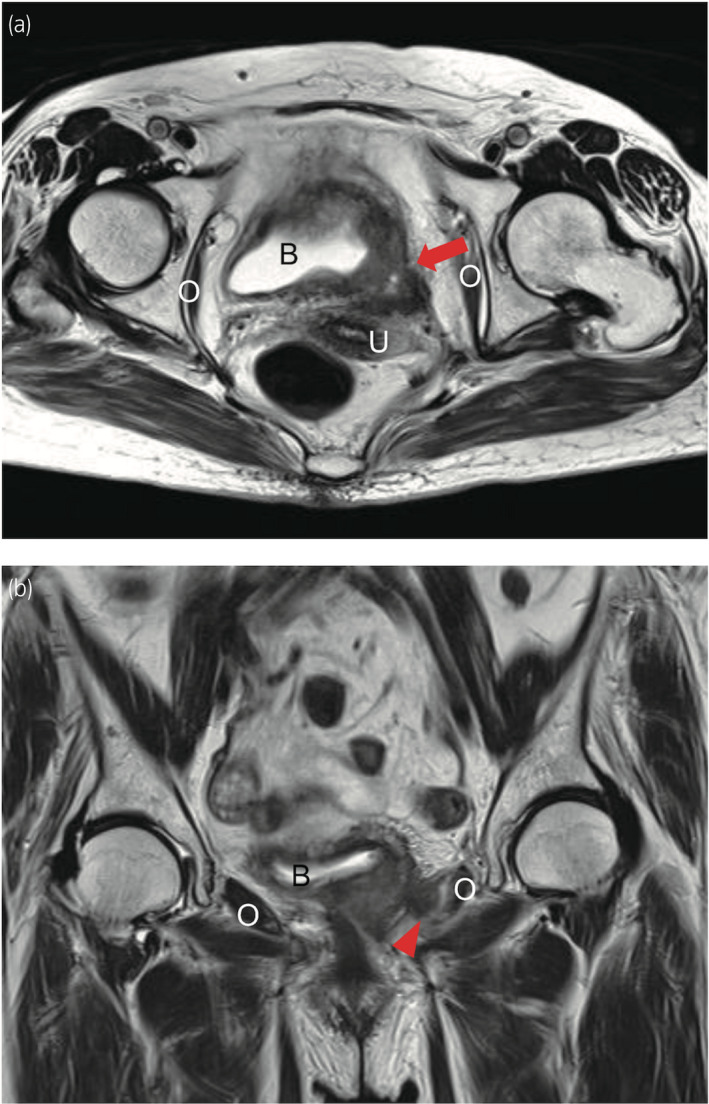
MRI findings of bladder cancer after radiochemotherapy. (a) Axial view: bladder cancer extends to the perivesical tissue (arrow). (b) Coronal view: bladder cancer attaches to the left internal obturator muscle (arrowhead). B, bladder; O, internal obturator muscle; U, uterus.

A 79‐year‐old woman, complaining of pain on urination, was referred to our department. She had a surgical history of left nephrectomy for renal tuberculosis 64 years earlier and the anterior and posterior TVM procedure for POP 8 years earlier. Cystoscopy showed diffuse erythematous bladder mucosa, and urinary cytology was positive for cancerous cells. MRI and CT findings showed neither bladder tumors nor metastatic lesions. Cold‐cup biopsies from the bladder mucosa were taken; the pathological analysis indicated a high‐grade, flat, non‐muscle invasive urothelial carcinoma. CIS of the bladder was diagnosed, and weekly intravesical BCG therapy was administered. After five injections, intravesical BCG therapy was discontinued because of severe pain. Follow‐up MRI showed a bladder tumor (4.5 cm in diameter) in the trigone area, which had infiltrated the perivesical tissue. Upon vaginal examination, a hard mass was palpable at the anterior vaginal wall. Right ureterostomy was established before the right ureteral orifice obstruction. Despite radiochemotherapy (nedaplatin 30 mg/m^2^ weekly and external beam radiotherapy 39.6 Gy/22 Fr), the tumor only decreased from 4.5 cm to 3.5 cm in size on MRI (Fig. 1a,b). Chronic cystitis persisted from admission and antibacterial treatment was necessary on occasion; however, the degree of inflammation by blood test recovered to an almost normal level.

**Fig. 2:**
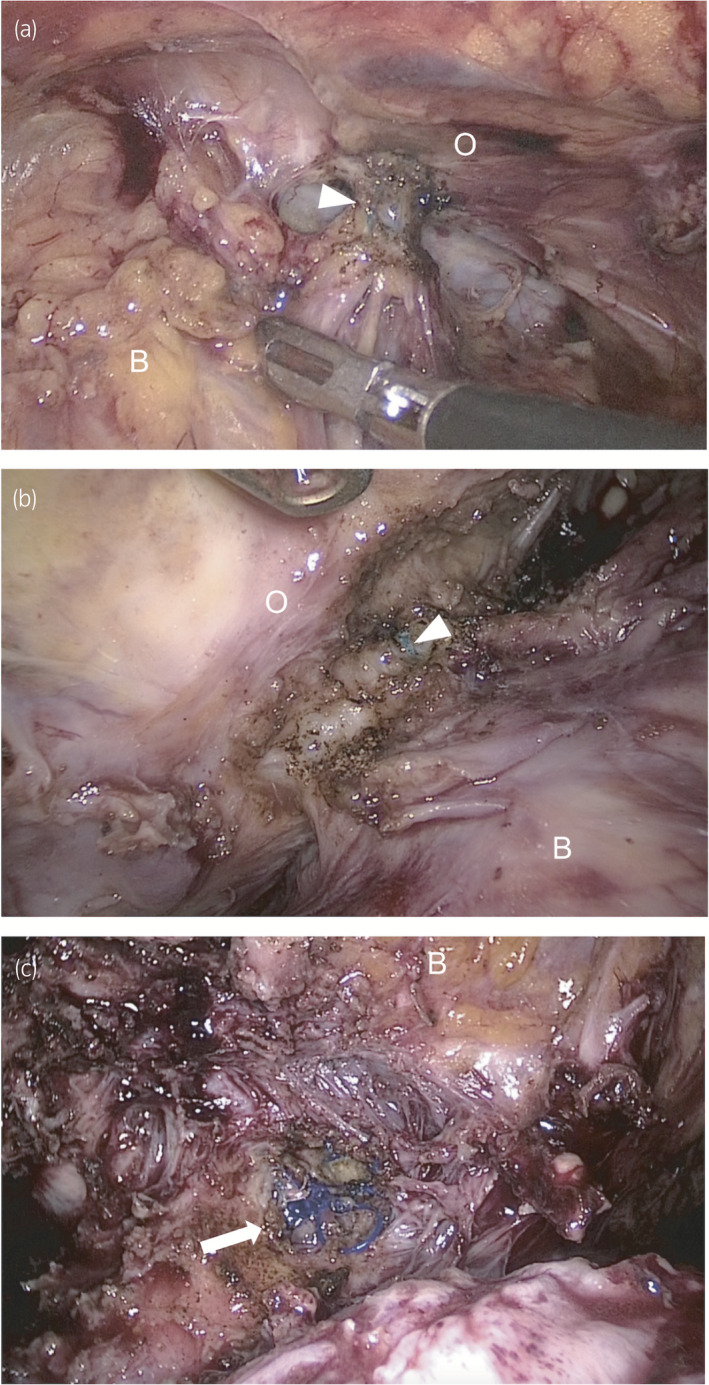
Intraoperative view of LRC. (a) The right side of the retropubic space. The anterior mesh arm (arrowhead) penetrating the right internal obturator muscle is observed. (b) The left side of the retropubic space. Extensive and strong adhesion between the bladder wall and internal obturator muscle was indicated around the left anterior mesh arm (arrowhead). (c) The anterior vaginal fornix. Sutures that fixed the anterior mesh to the uterine cervix were observed (arrow). B, bladder; O, internal obturator muscle.

**Fig. 3:**
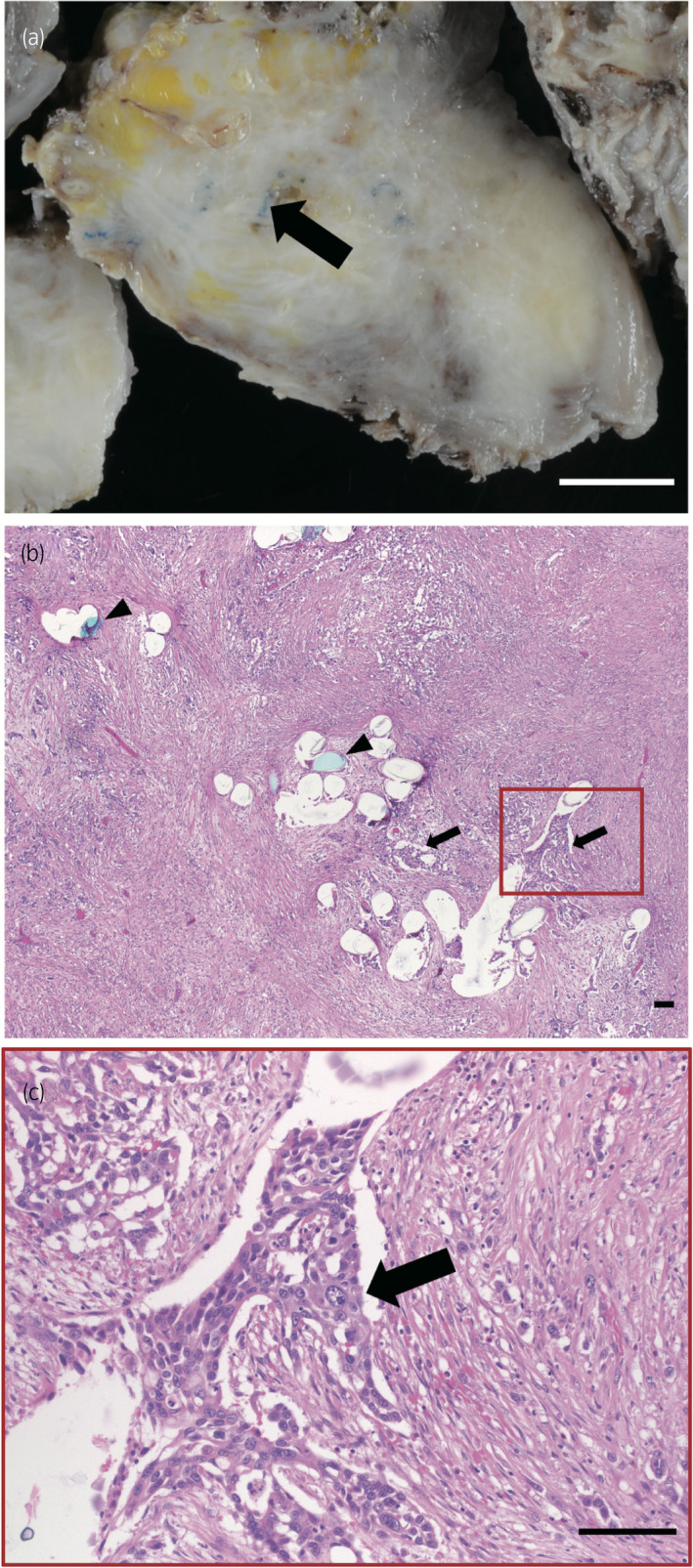
Findings of pathology. (a) Cut surface of the resected urinary bladder. Elastic firm tumor surrounding the blue mesh (arrow). Scale bar: 10 mm. (b, c) Histopathology of the urinary bladder tumor (hematoxylin‐eosin stain). (b) Bird’s eye view shows carcinoma cells (arrows) infiltrating the polypropylene mesh (arrowheads). Scale bar: 100 μm. (c) High magnification of the red square section in (b), showing high‐grade urothelial carcinoma cells (arrow). Scale bar: 100 μm.

**Fig. 4:**
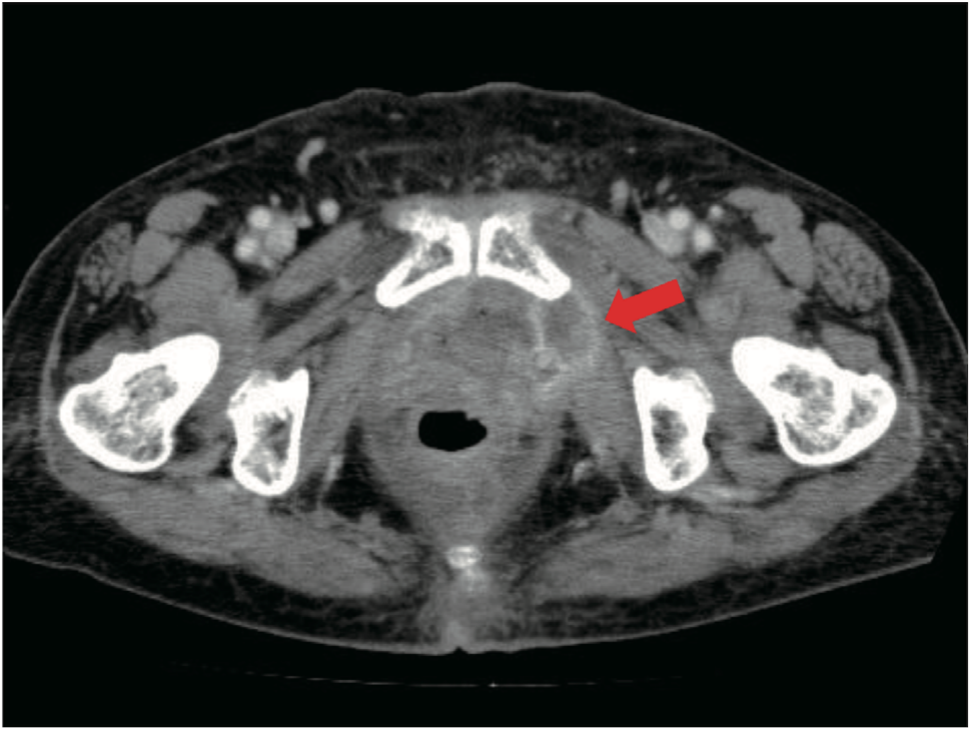
Axial view of CT image at 6 weeks postoperatively. Tumor has recurred in the left internal obturator muscle (arrow).

LRC was performed after a month of radiochemotherapy because of persistent severe bladder pain. In the right retropubic space, we identified the right anterior mesh arm of the TVM procedure penetrating the right internal obturator muscle (Fig. 2a). There was an extensive and strong adhesion between the bladder wall and left internal obturator muscle around the left anterior mesh arm (Fig. 2b). Although intraoperative rapid pathological diagnosis did not be performed, the left internal obturator muscle around the mesh arm was widely resected to excise the tumor. At the anterior vaginal fornix, sutures fixing the anterior mesh to the uterine cervix were visualized (Fig. 2c). From the anterior vaginal fornix, the anterior vaginal wall was incised, and the bladder was removed. The uterus was resected at the cervix, and the anterior vaginal wall defect was closed. The posterior mesh was preserved without exposure. Bilateral obturator lymph node dissection was also performed. The operation time was 9 h 59 min, estimated blood loss was 100 mL, and no perioperative complications occurred. The cut surface of the resected specimen showed a firm fibrotic tumor measuring 5 cm × 3 cm, which contained the mesh (Fig. 3a). Histologic sections revealed high‐grade urothelial carcinoma involving the entire bladder wall and infiltrating the perivesical tissue and vagina; additionally, the mesh was embedded within the firm cancer tissue (Fig. 3b,c). No obturator lymph node metastasis was indicated. Pelvic pain initially improved but recurred 6 weeks later. CT revealed a tumor recurrence in the left internal obturator muscle (Fig. 4) and sacral metastasis. Consequently, the patient died 5 months postoperatively.

## Discussion

We report a rare case of bladder cancer rapidly invading the pelvic wall muscle in a patient after a TVM procedure. The carcinogenicity of synthetic mesh in humans has been debated. Two cases of squamous cell carcinoma have been reported in postoperative polyester mesh surgeries of inguinal hernias that might have been caused by long‐term chronic infection.^8^ Although chronic inflammation due to implanted material was an etiological factor of carcinogenicity, there is no solid evidence of the carcinogenicity of polypropylene,^9^, ^10^, ^11^ which was the material used in the TVM procedure. In our case, the bladder cancer was a urothelial carcinoma and initially occurred in the bladder mucosa. Therefore, it is unlikely that the bladder cancer was caused by the mesh. However, pathological findings showed desmoplastic high‐grade urothelial carcinoma infiltrating around the mesh. The effect of mesh on the progression of existing cancers is not well known. There is only one case report of ovarian serous carcinoma recurrence in a synthetic mesh; the authors speculated that chronic inflammation caused by the insertion of polypropylene mesh affected tumor progression.^12^ We speculate that the scar tissues around the mesh may have become an abnormal anatomical pathway, and newly developed bladder cancer may have spread along the mesh from the left internal obturator muscle.

The use of LRC in a patient who has undergone a TVM procedure remains unclear. Usually, radical cystectomy in female patients is performed by incising the vaginal wall at the posterior vaginal fornix and resecting the uterus *en bloc* with the bladder.^13^ By incising the vaginal wall at the anterior vaginal fornix, we could resect the bladder and the anterior mesh without exposing the posterior mesh.

Our case suggests that mesh can become an abnormal anatomical pathway for tumor invasion. Both presurgical screening and long‐term postoperative follow‐up are needed in patients after a TVM procedure for early detection and treatment of bladder cancer before the tumor invades along the mesh.

## Conflict of interest

The authors declare no conflict of interest.

## References

[1] Takahashi S , Obinata D , Sakuma T *et al*. Tension‐free vaginal mesh procedure for pelvic organ prolapse: a single‐center experience of 310 cases with 1‐year follow up. Int. J. Urol. 2010; 17: 353–8. 20202001 10.1111/j.1442-2042.2010.02469.x

[2] Kato K , Suzuki S , Yamamoto S *et al*. Clinical pathway for tension‐free vaginal mesh procedure: evaluation in 300 patients with pelvic organ prolapse. Int. J. Urol. 2009; 16: 314–7. 19207605 10.1111/j.1442-2042.2008.02249.x

[3] Takeyama M . Basic procedures in tension‐free vaginal mesh operation for pelvic organ prolapse. Int. J. Urol. 2011; 18: 555–6. 21711397 10.1111/j.1442-2042.2011.02805.x

[4] Ganatra AM , Rozet F , Sanchez‐Salas R *et al*. The current status of laparoscopic sacrocolpopexy: a review. Eur. Urol. 2009; 55: 1089–103. 19201521 10.1016/j.eururo.2009.01.048

[5] Sarlos D , Kots L , Ryu G , Schaer G . Long‐term follow‐up of laparoscopic sacrocolpopexy. Int. Urogynecol. J. 2014; 25: 1207–12. 24700356 10.1007/s00192-014-2369-y

[6] Casey RG , Catto JW , Cheng L *et al*. Diagnosis and management of urothelial carcinoma in situ of the lower urinary tract: a systematic review. Eur. Urol. 2015; 67: 876–88. 25466937 10.1016/j.eururo.2014.10.040

[7] Babjuk M , Burger M , Compérat EM *et al*. European Association of Urology guidelines on non‐muscle‐invasive bladder cancer (TaT1 and carcinoma in situ) – 2019 update. Eur. Urol. 2019; 76: 639–57. 31443960 10.1016/j.eururo.2019.08.016

[8] Birolini C , Minossi JG , Lima CF , Utiyama EM , Rasslan S . Mesh cancer: long‐term mesh infection leading to squamous‐cell carcinoma of the abdominal wall. Hernia 2014; 18: 897–901. 23604537 10.1007/s10029-013-1083-x

[9] Moalli P , Brown B , Reitman MT , Nager CW . Polypropylene mesh: evidence for lack of carcinogenicity. Int. Urogynecol. J. 2014; 25: 573–6. 24614956 10.1007/s00192-014-2343-8 PMC5139346

[10] Linder BJ , Trabuco EC , Carranza DA , Gebhart JB , Klingele CJ , Occhino JA . Evaluation of the local carcinogenic potential of mesh used in the treatment of female stress urinary incontinence. Int. Urogynecol. J. 2016; 27: 1333–6. 26864666 10.1007/s00192-016-2961-4

[11] Altman D , Rogers RG , Yin L , Tamussino K , Ye W , Iglesia CB . Cancer risk after midurethral sling surgery using polypropylene mesh. Obstet. Gynecol. 2018; 131: 469–74. 29420401 10.1097/AOG.0000000000002496

[12] Grin L , Namazov A , Gemer O . Ovarian serous carcinoma in synthetic mesh: a rare case report and review of the literature. J. Obstet. Gynaecol. Res. 2019; 45: 1205–8. 30950130 10.1111/jog.13963

[13] O’Neil B , Scarpato KR , Chang SS . Radical cystectomy in female patients. In: Smith JA , Howards SS , Preminger GM , Dmochowski RR (eds). Hinman's atlas of urologic surgery, 4th edn. Elsevier, Philadelphia, 2017; 362–7.

